# Practical Departments

**Published:** 1904-10-01

**Authors:** 


### PRACTICAL DEPARTMENTS.

### A RAILWAY HAMMOCK.

On a previous occasion we drew attention to Miss Mary Horne's (Castle Grounds, Devizes) bath-chair improvements, which consist of easily adjustable hammock arrangements, whereby the chair can be converted into a comfortable invalid couch. Miss Horne is now endeavouring to introduce her hammock for the use of invalids travelling by rail, who cannot afford a regular invalid carriage. She is endeavouring to induce railway companies to fix some rings permanently to at least one third-class carriage on every train, so that her system might be put into use at short notice. There can be no doubt that the hammock would afford much comfort to the invalid, and we hope that she will succeed in introducing it for general use. She contends that if her system is rendered available it will be possible to move hospital and private patients at a much earlier stage in their convalescence, thus releasing accommodation for other cases in institutions, besides expediting the recovery of patients requiring change of air.

